# Segmental lumbar mobility in individuals with low back pain: in vivo assessment during manual and self-imposed motion using dynamic MRI

**DOI:** 10.1186/1471-2474-8-8

**Published:** 2007-01-29

**Authors:** Kornelia Kulig, Christopher M Powers, Robert F Landel, Hungwen Chen, Michael Fredericson, Marc Guillet, Kim Butts

**Affiliations:** 1 Department of Biokinesiology and Physical Therapy, University of Southern California, 1540 East Alcazar St., CHP-155, Los Angeles, CA 90089, USA; 2 Department of Functional Restoration, Division of Sports Medicine, Stanford University, Stanford, CA 94305, USA; 3 Department of Radiology, Stanford University, Stanford, CA 94305, USA

## Abstract

**Background:**

Altered spinal mobility is thought to be related to current or past episodes of low back pain; however evidence of that relationship in younger subjects has not been established. The purpose of this study was to compare lumbar segmental mobility in asymptomatic and symptomatic subjects during posterior to anterior (PA) manual spinal mobilization and a self-initiated prone press-up (PU) maneuver. We hypothesized that persons with central low back pain would have an altered lumbar segmental mobility pattern compared to those without pain.

**Method:**

Forty-five individuals (age 32.1 ± 8.5) with non-specific low back pain and 20 persons (age 31.1 ± 7.0) without low back pain participated. Each subject underwent dynamic imaging of the lumbar spine during a PA mobilization procedure and while performing a PU. Segmental motion was quantified as the change in the intervertebral angle between the resting and end-range vertebral positions.

**Results:**

The symptomatic group had a larger percentage of subjects with evidence of single level segmental hypermobility than the asymptomatic group during the PA (40.0% vs. 5%) and PU (26.7% vs. 15%) procedures. Single lumbar motion-segment analysis revealed hyper-mobility in symptomatic subjects at L5 – S1 (Chi-square = 10.0, p ≤ 0.01) and L4 – L5 (Chi-square = 4.18, p ≤ 0.05) during the PA test.

**Conclusion:**

Persons with non-specific low back pain have a tendency to demonstrate single level lumbar segmental hypermobility when compared to age specific asymptomatic subjects.

## Background

Back pain and its association with altered functional ability can significantly affect an individual's quality of life [1,2]. Prevalence of low back pain throughout the lifespan has been reported to be between 13 and 85% [3-7]. The point prevalence of back pain varies with age [7]. Back pain is considered the plague of middle-age; however, it can certainly begin its manifestation in the younger individuals. [8] Even though the resolution of acute symptoms seems faster in younger person, one of four may continue having symptoms 12 weeks after the onset of symptoms resulting in pain and cost to economy [8,9].

A painful low back does not indicate a specific pathology but may be related to restricted, excessive, or poorly controlled lumbar motion [10-12] Altered mobility can be characterized as general (mobility of the trunk as a whole) or segmental (between two consecutive vertebra) [13]. Assessment of spinal mobility often guides the non-surgical treatment approach. For example, a patient with restricted spinal motion may receive interventions directed at improving intervertebral mobility whereas an individual with symptoms and an excessively mobile spine may receive active and/or resistive exercises to provide control of aberrant movements (i.e., "stabilization" exercises) [14].

General trunk mobility has been quantified in the clinical setting using methods of linear or angular displacement [15]. These tests, however, do not capture the existence of altered mobility at a single segment or unique patterns of aberrant segmental mobility. Clinical methods to evaluate segmental motion include manual application of a posterior to anterior (PA) force on the vertebral spinous process [16] or palpation of movement between spinous processes during flexion-extension of the trunk [16]. In both cases, the amount of motion, or resistance to force, is assessed using subjective categories of hypomobile, normal, or hypermobile. Presence, absence or change in pain resulting from the test is also noted.

It is not clear if persons with current back pain, or those with a history of frequent episodes of back pain, have altered segmental spinal mobility. Previous studies have attempted to correlate spinal mobility to clinical symptoms but have not been able to clearly establish a relationship [17-22]. Structural spine heterogeneity in the human population, age-related changes in spinal motion and experimental methodology are among just few of the confounding factors that have impeded previous attempts to establish a relationship between lumbar spine mechanics and low back pain. In addition, previous literature has focused predominantly on subjects in the middle and later stages of life, as symptoms in younger subjects are typically shorter in duration [23].

Quantity and pattern of segmental mobility may certainly vary with the type of movement tested, instructions to the subjects and their willingness to participate. For example, a self initiated test, such as a press-up (PU) maneuver may be influenced by the subject's symptoms, thereby limiting spinal segmental motion. In contrast, application of a manual PA force to a lumbar spinous process sufficient in amount to reach end-range may produce more motion since it's not self-limited by pain. Therefore, quantifying motion under these two conditions may provide a more comprehensive assessment of segmental mobility in persons with low back pain. Segmental motion of the lumbar spine during manual PA assessment has been investigated [24-27] and we are not aware of any studies that have assessed segmental mobility during a prone PU maneuver.

Using dynamic imaging techniques, the purpose of this study was to compare lumbar segmental mobility in asymptomatic and symptomatic subjects during PA spinal mobilization and a prone PU maneuver. We hypothesized that persons with central low back pain would have an altered lumbar segmental mobility pattern compared to those without pain.

## Methods

### Subjects

A total of 65 individuals between the ages of 18 and 42 participated in this study. The asymptomatic group was comprised of 20 healthy individuals, with no history of low back pain lasting more than 3 days or pain reported within the previous year (Table 1). The symptomatic subjects were recruited from a very busy University based general practice. Forty-five patients with non-specific central low back pain that worsened with extension, constituted the symptomatic group (Table 1). Based on the appearance of the fifth lumbar vertebrae, suggesting developmental variance one subject was excluded from further participation. An additional symptomatic subject was recruited to meet the expected subject population in this study. This analysis is a sub-protocol of a larger study thus the discrepancies in the size of the two study populations. On the day of testing, these subjects had perceived pain with standing spinal extension averaging 4.3 ± 1.9 on the Visual Analog Scale and their Oswestry Disability Scores range between minimal and moderate. The reported average duration of symptoms of the current episode of low back pain was 28 days. None of them were off work. Patients for whom the current episode of back pain lasted longer than 12 weeks were not included in this study.

The primary exclusion criteria for both groups were: 1) spinal malignancy, 2) cardiovascular disease, 3) evidence of cord compression, 4) aortic aneurysm, 5) hiatal hernia, 6) uncontrolled hypertension, 7) spinal infection, 8) severe respiratory disease, 9) pregnancy, 10) abdominal hernia, 11) prior low back surgery, 12) gross spinal deformity, 13) spondylolisthesis, 14) known rheumatic joint disease, and 15) implanted biological devices that could interact with the magnetic field (i.e., pacemakers, cochlear implants, or ferromagnetic cerebral aneurysm clips). In addition to the above exclusion criteria, subjects in either group could not have any signs or symptoms related to nerve root or cord pathology. Therefore, subjects who demonstrated any of the following also were excluded: 1) radiating pain below the level of the buttock(s), 2) sensation changes in the lower extremities, 3) diminished reflexes, 4) lower extremity weakness, 5) urinary or fecal incontinence, and 6) increased peripheral pain with repeated lumbar extension.

### Instrumentation

As described in a previous publication, [27] dynamic imaging of the lumbar spine was performed using a vertically opened (double donut design) MRI system (0.5 Tesla, Signa SP, General Electric Medical Systems, Milwaukee, WI) with a 56 cm opening that allowed the examiner access to the subject during testing. This system was equipped with a pulse sequence programming environment and real-time interactive MRI capability.

Midline sagittal plane imaging of the spine was performed using a receive-only surface coil and an ultrafast spoiled GRASS (gradient recalled acquisition in the steady state) pulse sequence. Images were obtained at a rate of 1 per second using the following parameters: repetition time (TR): 200 ms; echo time (TE): 18 ms; number of excitations: 1.0; matrix: 256 × 256; field of view (FOV): 28 × 21 cm; and a 7 mm section thickness with an interslice spacing of 1 mm. [27] The surface coil was flexible and designed to expose the low back such that the examiner had direct access to palpate the lumbar spinous processes.

### Procedure

Prior to participation, informed consent was obtained as approved by the Institutional Review Boards of the University of Southern California and Stanford University. Two sets of images were acquired for each subject: 1) during the manual PA mobilization procedure and 2) during a PU maneuver. The order of testing was randomized for each subject.

**Figure 1:**
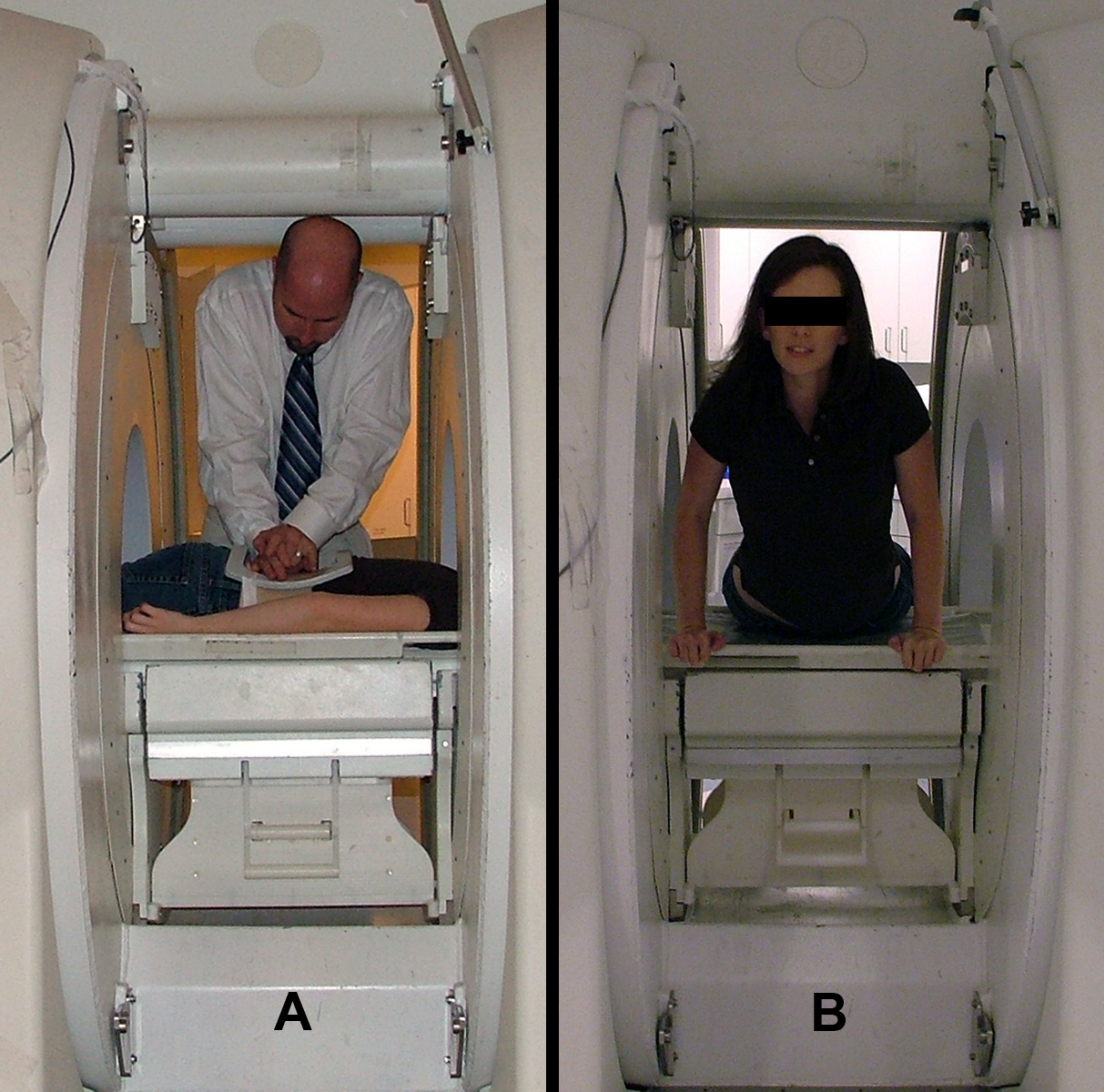
Subject and examiner positioned within the MRI system for (a) the segmental motion of the target lumbar segment during the posterior to anterior (PA) mobilization procedure and (b) the prone press-up PU maneuver. Each subject was situated such that the spine and torso were within the opening between the vertical magnets.

For the PA assessments, subjects were placed in the prone position on a sliding table within the MRI system, such that the spine and torso were within the opening between the vertical magnets (Figure 1a). For each of the two procedures, the surface coil was secured to the lumbar region using cloth tape. Careful care was taken to place the coil in the same region of the subject's low back. A small pillow was positioned under the subject's abdomen, thereby mimicking the clinical procedure.

To gain an insight into the posture of the lumbar spine in the starting prone position, we measured the angular position of the lumbar curvature with an inclinometer (Universal Inclinometer, OPTP, Minneapolis, MN). To do so, we placed an inclinometer at the following anatomical locations S2, L3 and L1.

After subjects were positioned within the MRI system, a series of sagittal plane "localizers" were obtained to ensure that the image plane captured the vertebral bodies and spinous processes of all lumbar vertebrae. Once the image plane was determined, continuous imaging (1 Hz) commenced.

Following a static sagittal view of the lumbar spine in the resting position, a PA force was applied to the spinous processes at each vertebrae starting caudally at L5 and moving cranially to L1. The force was directed anteriorly in a direction perpendicular to the tangent of the arc of the lumbar lordosis. Sufficient force was applied to induce movement to the end of the available range of segmental motion and was comparable in magnitude to that of a "grade IV" as defined by Maitland. [16] The force was applied slowly (over approximately a 1–2 second period) and held at end range for at least five seconds. Release of the force also occurred slowly (1–2 seconds) before applying force to the next vertebral level. Once the examiner released the force on L1, and a clear resting position was observed, imaging was terminated.

All PA mobilizations were performed by a physical therapist with 15 years of manual therapy experience. A second investigator viewed the images on the MRI console to assure that the forces were being applied to the correct vertebral level. If an error was observed, imaging was repeated (this has occurred in three instances).

For the PU assessment, subjects were placed in the prone position on the sliding table, such that the spine and torso were within the opening between the vertical magnets and the head and arms were outside of the MRI system (Figure 1b). For this procedure, the patient's positioning within the magnet was perpendicular to the position during the PA mobilization testing. The surface coil was again secured to the lumbar region using cloth tape. A small pillow was positioned under the subject's abdomen for comfort. Following the subject's new positioning within the MR system, the sagittal plane "localizers" were repeated to ensure that the image plane captured the vertebral bodies and spinous processes of all lumbar vertebrae. Once the image plane was determined, continuous imaging (1 Hz) commenced. After the acquisition of a static sagittal view of the lumbar spine in the resting position, subjects placed their hands in front of them and arched their back as far as possible within pain tolerance (Figure 1b). Subjects were instructed to hold the end press-up position for a count of 10 and slowly lower the trunk to the resting position. Upon returning to the prone position, imaging was terminated.

### Data analysis

Prior to analysis, all images were transferred from the MRI system console to a Macintosh G3 computer (Apple Computer, Cupertino, CA). For purposes of this study, only the images at rest and at the end-range of segmental motion were analyzed. All data sets were again screened for quality of images and for pathology. Consequently, all images were deemed appropriate for further analysis. During screening, we noticed motion artifacts in the images where motion was expected. This helped in choosing the images for analysis. As the beginning of motion was readily observable, we chose the resting image for analysis at lest 5 images before motion was observed. The end of motion image was also chosen based clarity of the images, suggesting no motion. To assure ourselves that this assumption was correct, we have randomly selected 5 studies and have digitized three consecutive images deemed to be at the end of the range. This experiment assured us that our prior choice of the final image was correct.

**Figure 2:**
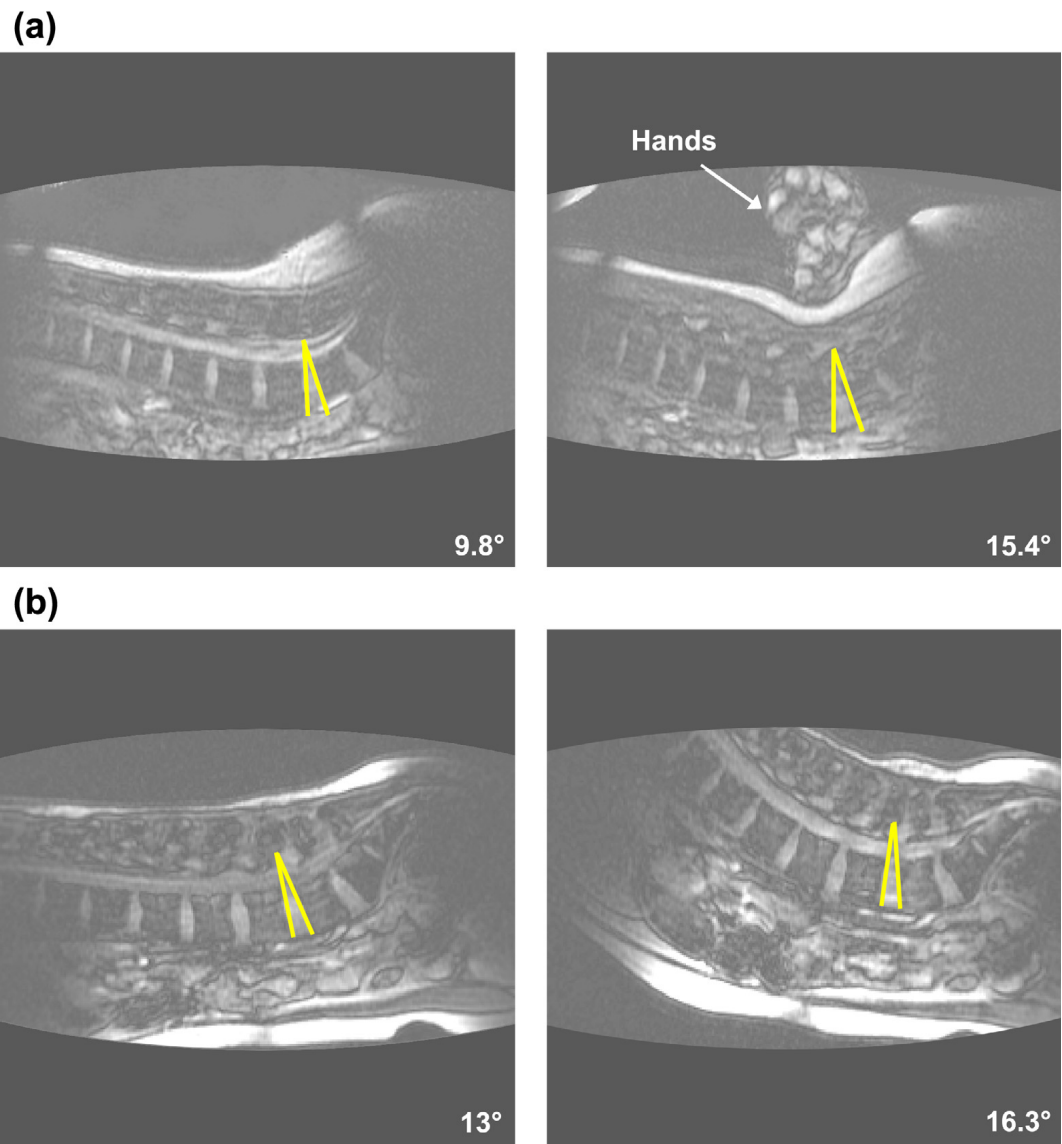
Measurements of intervertebral displacement resulting from the posterior to anterior (PA) mobilization and the prone press-up (PU) maneuver. The intervertebral (segmental) angle was measured as the angle formed by lines defining the endplates of adjacent vertebrae. Segmental lumbar displacement was defined as the difference in the intervertebral angle between the resting position (left) and intervertebral angle from the end range image (right). The arrow in Figure 2a identifies the hand of the examiner performing the PA mobilization.

Lumbar segmental motion in the sagittal plane was quantified by measuring the intervertebral angle which was defined as the angle formed by lines delineating adjacent vertebral endplates (Figure 2) [27]. Segmental lumbar motion was defined as the difference between the intervertebral angles measured from the resting and the end-range images. An increase in intervertebral motion from the resting to the end-range position was indicative of spinal extension. Conversely a decrease in the intervertebral motion was indicative of spinal flexion. The superior vertebra was used to define the target functional spinal unit. For example, a PA force applied to L4 was identifying the function spinal unit of L4-L5.

The same investigator, blinded to the group assignment, made all measurements using the National Institutes of Health (NIH) software (Bethesda, MD). To establish the intra-tester reliability of angular measurements, dynamic MR images obtained from 5 healthy volunteers were measured on two separate occasions. Intraclass Correlation Coefficients (ICC) were found to be excellent, ranging from 0.95 to .99 for all subjects. The Standard Error of Measurement was determined to be between 0.4 and 0.66 degrees.

### Statistical analysis

For the PA analyses only the data from the motion-segment associated with the PA was used for the analysis. The data for both groups was screened for normality of distribution using Wilks-Shapiro W statistics. The symptomatic group did not exhibit normal distribution; therefore non-parametric statistics were used for further analyses.

To determine the distribution of subjects outside of the normal range of segmental mobility, the norms were established based on the asymptomatic group's mean value ± 2 SD (see Tables 2 and 3). Individuals demonstrating segmental motion greater than 2 standard deviations range will be considered hypermobile while those demonstrating less than 2 standard deviations range were considered to be hypomobile. Overall frequency count will determine number of segments outside of normal range in each group of subjects. A Chi-square analyses of data at each motion- segment, comparing groups (control, symptomatic) and mobility (normal, non-normal) will be performed using SPSS statistical software (SPSS Inc., Chicago, IL) with a significance level of p < 0.05.

## Results

There was no significant difference in the starting prone position of the pelvis and the lumbar spine, between the symptomatic and asymptomatic groups. The average angular position relative to horizontal was 19.1 ± 1.2° at S1, 8.8 ± 2.9° at L3 and 2.3 ± 2.6° at L1.

**Figure 3:**
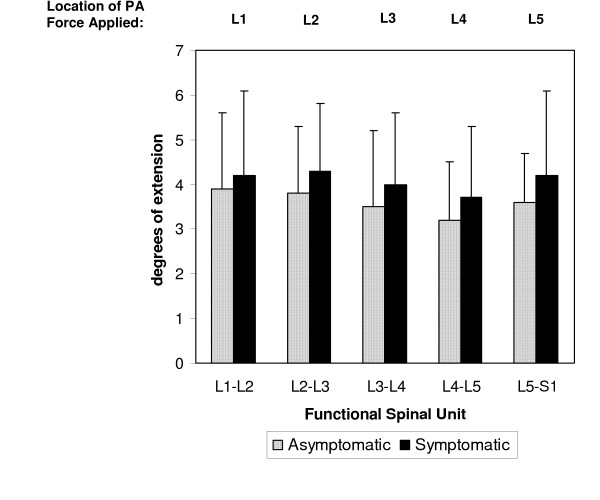
Mean segmental motion of the target lumbar segment during the posterior to anterior (PA) mobilization procedure. Error bars represent 1 SD.

The application of the PA force during the mobilization procedure resulted in extension at each targeted lumbar segment (Figure 3). The largest amount of motion occurred at L1-L2 in asymptomatic subjects (3.9 ± 1.7 degrees) and at L2-L3 in symptomatic subjects (4.3 ± 1.5 degrees). The least amount of motion was measured at L4-5 for both asymptomatic and symptomatic subjects (3.2 ± 1.3 degrees and 3.7 ± 1.6 degrees, respectively).

**Figure 4:**
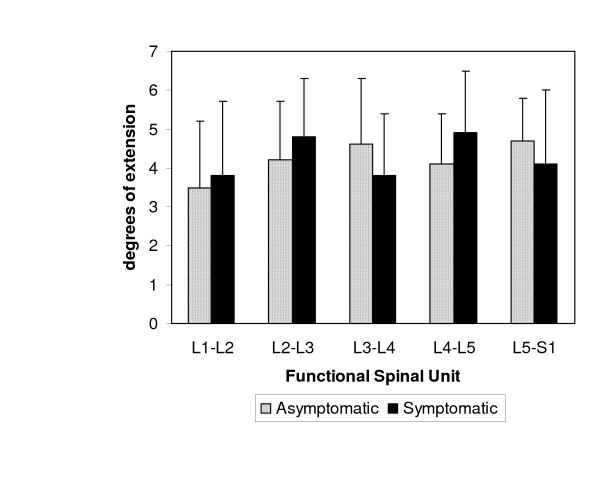
Mean segmental motion of the lumbar segments during the prone press-up (PU) maneuver. Error bars represent 1 SD.

Similar to the PA mobilization procedure, the PU maneuver resulted in extension at each segment (Figure 4). The greatest amount of motion occurred at L5-S1 in asymptomatic subjects (4.7 ± 1.1 degrees) and at L4-L5 in symptomatic subjects (4.9 ± 1.6 degrees). The least amount of motion was measured at L1-L2 for both asymptomatic and symptomatic subjects (3.5 ± 1.7 degrees and 3.8 ± 1.9 degrees, respectively).

The frequency analysis revealed that 40.0% of symptomatic subjects demonstrated hyper-mobility at one or more motion-segments during the PA assessment, while 26.7% of symptomatic subjects demonstrated hyper-mobility at one or more motion-segments during the PU maneuver. In contrast, only one subject (5%) demonstrated hyper-mobility at one motion segment during the PA assessment, while 3 subjects (15%) demonstrated hyper-mobility at one motion-segment during the PU maneuver. Individual lumbar motion-segment analysis revealed hyper-mobility in symptomatic subjects at L5 – S1 (Chi-square = 10.0, p ≤ 0.01) and L4 – L5 (Chi-square = 4.18, p ≤ 0.05) (Table 2).

The number of subjects demonstrating hypo-mobility was low in both groups. For example, during the PA assessment only 4.4% of symptomatic and 10% of asymptomatic subjects demonstrated hypo-mobility at one motion-segment. Similarly, 8.9% of symptomatic and 5% of asymptomatic subjects demonstrated hypo-mobility at one motion-segment during the PU maneuver (Table 2 and Table 3).

## Discussion

We hypothesized that there would be a difference in lumbar segmental mobility between persons with and without low back pain. We found more cases of increased lumbar segmental mobility, in subjects with low back pain than those who are pain-free, when tested manually. These hyper-mobilities were observed at the L4-L5 and L5-S1 motion-segments.

The highest incidence of hypermobility in the symptomatic group was observed at L5-S1 during the PA procedure and at L4-L5 during the PU. When the frequencies of hypermobility at L5-S1 and L4-L5 were combined, they constituted 56.5% and 66.7% of all levels for the PA and PU procedures, respectively. This analysis suggests that a relatively high percentage of subjects in the symptomatic group demonstrated tendency towards hypermobility, especially at the L4-L5 and L5-S1 motion-segments. The tendency towards segmental hypermobility in our young symptomatic subjects is similar to that identified by Dvorak et al [28].

The quantities of intervertebral motions imparted by the two tests were different. Overall, the PU test produced slightly more movement at four of the five motion-segments than the PA test. Suggesting, that the subjects were willing to extend their back and pain was not limiting their lumbar extension in the prone position. Interestingly, it was not the self-administered PU test, but the manual PA test, that identified the hypermobile segments. It is possible that the participants were able to control the motion during the PU maneuver and the PA imparted the intervertebral motion passively. This observation suggests that the PA procedure allows for identifying hypermobilities.

Increased motion (hyper-mobility) can be linked with recent disc injury, early disc degeneration or skeletal trauma. Subjects with skeletal trauma were excluded from this study. Hence relating intervertebral mobility to the condition of the disc is reasonable. As the mean age of the subjects in our study was in the early thirties, it is likely that their intervertebral discs were relatively normal, or at the very least, in the early stages of degeneration. The general trends in later life are associated with further disc degeneration and consequent joint degenerations leading to decreased motion (hypo-mobility). Therefore, the age of the subjects in this study may have had an influence on measured segmental mobility.

The observation made from this study need to be viewed within its limitations. We relied on the experience of the operator (physical therapist) to apply sufficient about of force to produce end range at a motion-segment. We might have had introduced systematic bias into the PA experiment, by applying force from caudal to cranial, instead of randomizing the location of the force application. We studied a limited age range and are not able to compare between younger and older age groups.

## Conclusion

This study quantified in vivo segmental spinal mobility during a PA mobilization procedure and during a prone PU maneuver in persons with and without low back pain. The PA procedure identified hypermobilities at the L4-L5 and L5-S1 motion segments in the symptomatic group.

## Completing interests

The author(s) declare that they have no competing interests.

## Authors' contributions

KK, CMP, and RFL were responsible for the design of this study. CMP secured funding for this study. HW performed the data processing and analysis. MF and MG contributed to data collection and data interpretation. KB provided MRI expertise.

All authors read and approved the final manuscript.

## Pre-publication history

The pre-publication history for this paper can be accessed here:



## Figures and Tables

**Table 1:** Anthropometric, gender and age characteristic of patients (symptomatic) and subjects (asymptomatic).

	Symptomatic		Asymptomatic	
	
	Male (N = 18)	Female (N = 27)	Male (N = 12)	Female (N = 8)
Age (years)	34.1 ± 8.6	30.0 ± 8.3	30.9 ± 6.4	31.3 ± 7.6
Body Mass (kg)	80.8 ± 11.9	64.2 ± 10.4	82.2 ± 13.6	57.0 ± 5.5
Body Height (cm)	180.1 ± 9.9	168.9 ± 7.2	177.5 ± 8.2	167.3 ± 4.8

**Table 2:** Mean and SD of vertebral displacement (degrees), for asymptomatic subjects, resulting from the PA testing procedure (first row). Values for vertebral displacement above (second row) and below (third row) two standard deviations (2SD) from the mean of asymptomatic subjects are in bold. Number of subjects with vertebral displacement above and below two standard deviations (2SD) from the asymptomatic group mean is listed bellow. Note that 1 subject in the asymptomatic group and 4 in the symptomatic group exhibited vertebral displacement outside of the 2SD range.

Motion-segment	L1 – L2	L2 – L3	L3 – L4	L4 – L5	L5 – S1	Number of subjects with at least one level above 2SD or below 2SD (%)
Mean (SD) vertebral displacement [degrees] for asymptomatic subjects	3.7 (1.4)	3.8 (1.4)	3.5 (1.7)	3.2 (1.3)	3.6 (1.1)	
+2SD [degrees]	**6.5**	**6.5**	**6.9**	**5.8**	**5.8**	
Asymptomatic (N = 20)	1	0	0	0	0	1 (5%)
Symptomatic (N = 45)	4^c,d^	2^c,d^	2	4^c^	9	18 (40%)

-2SD [degrees]	**0.9**	**0.9**	**0.0**	**0.9**	**1.3**	
Asymptomatic (N = 20)	0	1^a^	1^a^	1	0	2 (10%)
Symptomatic (N = 45)	2^b^	0	0	0	2^b^	2 (4.4%)

Chi- square				10.0 p < 0.01	4.18 p < 0.05	

^a ^includes subject 164; ^b ^includes subjects 54 and 173; ^c ^includes subject 1951; ^d ^includes subject 1955

**Table 3:** Mean and SD of vertebral displacement, for asymptomatic subjects, resulting from the PU maneuver (first row). Values for vertebral displacement above (second row) and below (third row) two standard deviations (2SD) from the mean of asymptomatic subjects are in bold. Number of subjects with vertebral displacement above and below two standard deviations (2SD) from the asymptomatic group mean is listed bellow. Note that 1 subject in the asymptomatic group and 1 in the symptomatic group exhibited motion outside of the 2SD range. There was no difference, between groups, in number of subjects exhibiting vertebral displacement outside the ± 2SD from the mean of the asymptomatic subjects.

Motion-segment	L1 – L2	L2 – L3	L3 – L4	L4 – L5	L5 – S1	Number of subjects with at least one level above 2SD or below 2SD (%)
Mean (SD) segmental motion [degrees] for asymptomatic subjects	3.4 (1.6)	4.1 (2.5)	4.6 (2.2)	4.1 (1.9)	4.7 (2.3)	
+2SD [degrees]	**6.6**	**9.1**	**9.0**	**7.9**	**9.3**	
Asymptomatic (N= 20)	0	2^a^	0	1^a^	1	3 (15%)
Symptomatic (N = 45)	5^b^	1^b^	0	5	2	12 (26.7%)

-2SD [degrees]	**0.2**	**-0.9**	**0.4**	**0.3**	**0.1**	
Asymptomatic (N = 20)	0	0	0	1	0	1 (5%)
Symptomatic (N = 45)	1	0	2	0	1	4 (8.9%)

^a ^includes subject 1891; ^b ^includes subjects 2012
